# Genomic evaluation of feed efficiency component traits in Duroc pigs using 80K, 650K and whole-genome sequence variants

**DOI:** 10.1186/s12711-018-0387-9

**Published:** 2018-04-06

**Authors:** Chunyan Zhang, Robert Alan Kemp, Paul Stothard, Zhiquan Wang, Nicholas Boddicker, Kirill Krivushin, Jack Dekkers, Graham Plastow

**Affiliations:** 1 Department of Agricultural, Food and Nutritional Science, University of Alberta, Edmonton, AB T6G 2R3 Canada; 2 Genesus Inc., Oakville, MB R0H 0Y0 Canada; 3 Department of Animal Science, Iowa State University, Ames, IA 50011 USA

## Abstract

**Background:**

Increasing marker density was proposed to have potential to improve the accuracy of genomic prediction for quantitative traits; whole-sequence data is expected to give the best accuracy of prediction, since all causal mutations that underlie a trait are expected to be included. However, in cattle and chicken, this assumption is not supported by empirical studies. Our objective was to compare the accuracy of genomic prediction of feed efficiency component traits in Duroc pigs using single nucleotide polymorphism (SNP) panels of 80K, imputed 650K, and whole-genome sequence variants using GBLUP, BayesB and BayesRC methods, with the ultimate purpose to determine the optimal method to increase genetic gain for feed efficiency in pigs.

**Results:**

Phenotypes of average daily feed intake (ADFI), average daily gain (ADG), ultrasound backfat depth (FAT), and loin muscle depth (LMD) were available for 1363 Duroc boars from a commercial breeding program. Genotype imputation accuracies reached 92.1% from 80K to 650K and 85.6% from 650K to whole-genome sequence variants. Average accuracies across methods and marker densities of genomic prediction of ADFI, FAT, LMD and ADG were 0.40, 0.65, 0.30 and 0.15, respectively. For ADFI and FAT, BayesB outperformed GBLUP, but increasing marker density had little advantage for genomic prediction. For ADG and LMD, GBLUP outperformed BayesB, while BayesRC based on whole-genome sequence data gave the best accuracies and reached up to 0.35 for LMD and 0.25 for ADG.

**Conclusions:**

Use of genomic information was beneficial for prediction of ADFI and FAT but not for that of ADG and LMD compared to pedigree-based estimates. BayesB based on 80K SNPs gave the best genomic prediction accuracy for ADFI and FAT, while BayesRC based on whole-genome sequence data performed best for ADG and LMD. We suggest that these differences between traits in the effect of marker density and method on accuracy of genomic prediction are mainly due to the underlying genetic architecture of the traits.

**Electronic supplementary material:**

The online version of this article (10.1186/s12711-018-0387-9) contains supplementary material, which is available to authorized users.

## Background

Feed is of major economic importance in pig production, accounting for 60 to 70% of total costs. The grow-finish phase accounts for the largest proportion of total feed, at about 75% [1]. Thus, improving grow-finish feed efficiency will significantly reduce production cost and increase profitability. Although intense selection for lean growth has improved feed efficiency dramatically in the past decades, with feed conversion ratio (FCR) values of 2.0 or less currently achievable [1], further improvements require direct measurement and selection on feed intake (FI) and other components of feed efficiency. This is especially the case for high-quality products with increased marbling, since fat deposition has a high genetic correlation with FI (0.37 [2]). However, the expense of recording FI on large numbers of selection candidates limits the opportunities of using this approach. Genomic selection (GS) or prediction is a promising approach to address this issue, since it allows for early selection among candidates without FI records, higher rates of genetic gain, and better management of inbreeding, compared with traditional selection based on pedigree and phenotype [3, 4].

GS has been widely applied in livestock breeding programs, using medium-to-high density single nucleotide polymorphism (SNP) panels [5]. The most successful implementation of GS is in dairy cattle, which has made it possible to reduce generation intervals and costs by eliminating progeny testing [6, 7]. Unlike dairy cattle, where the biggest impact is on reducing generation interval [8], the largest benefit for pigs is in increasing the accuracy of selection for traits such as feed intake. However, implementation of GS in pigs is still very limited [9–12], which might be due to the low monetary value of a boar compared to a dairy bull and the relatively low genomic prediction power for pigs in most breeding programs, due to not having access to large numbers of animals that have the necessary phenotype and genotype records compared to dairy cattle (primarily for Holsteins). It was anticipated that these limitations could be addressed by increasing the numbers of animals with quality phenotypes that are genotyped and the number of markers used (especially for markers that are in linkage disequilibrium (LD) with the underlying causative mutations) or by using the causative mutations themselves [7]. Using whole-genome sequence data is also expected to increase the accuracy of genomic prediction, since all or most of the causal mutations that underlie quantitative traits loci (QTL) are expected to be included in the data. Inclusion of the causal mutations is expected to increase the accuracy of genomic prediction across generations and even across breeds [7]. This was confirmed using simulated data [13–16] but, in practice, the use of imputed sequence data in cattle and chicken has shown little increase (0–3%) in the accuracy of genomic prediction [17–21]. Many factors can influence the accuracy of genomic prediction, including the genetic architecture of the traits, the statistical method applied [13, 22], marker density, LD between QTL and SNPs [16], effective population size [19, 23, 24], size of the reference population, relatedness of selection candidates with individuals in the training data [13, 22, 25], and imputation accuracy of marker genotypes [14]. The availability of higher density SNP panels and sequence information for pigs provided the opportunity to examine this for feed efficiency in a commercial Duroc breeding population.

Therefore, this study aimed at evaluating the accuracy of genomic prediction of feed efficiency component traits of average daily feed intake, average daily gain, ultrasound backfat depth, and loin muscle depth, using 80K and imputed 650K SNPs, as well as imputed whole-genome sequence variants. Three methods, GBLUP [26], BayesB [27, 28] and BayesRC [19], were compared to determine the best method and marker density for each trait. Possible factors that influence the accuracy of genotype imputation and genomic prediction were also discussed. The ultimate aim was to investigate the feasibility and optimal approach for using genomic information to increase genetic gain for feed efficiency in pigs.

## Methods

### Ethics statement

Data were collected at the Prairie Sun Research and Development Facility (Genesus Inc., Oakville, MB). All animals used in this study were raised under commercial production-like conditions and fed standard diets designed to exceed the pig’s requirements, as described previously [29]. The proposed work was reviewed by the University of Alberta Animal Care and Use Committee. No other specific permissions were required for the work, since the animals were cared for according to the Canadian Quality Assurance Program, which includes attention to animal health and well-being and is in line with the Canadian Council on Animal Care guidelines.

### Animals and data collection

A total of 1363 Duroc boars (from 63 sires and 439 dams) tested in 2014 were used for this study. At weaning, on average, two boars per litter were selected to create a group of 24 or 48 boars, depending on the number of litters weaned in a given week. The average genetic relationship among these 1363 individuals was about 0.12 based on pedigree information. The boars were placed in nursery pens at a stocking density of 24 per pen, with littermates split between the two pens when groups of 48 were stocked. At completion of the nursery phase (approximately 9 weeks of age), each group of boars was put into a single test pen (22 to 24 boars per pen) that was fitted with two electronic feeders per pen (IVOG, Insentec BV, Marknesse, the Netherlands). Boars from a nursery pen were kept together in the test pen. Following a 7-d acclimation period, feed intake was recorded in a test period of 14 weeks. Body weights were recorded at the beginning (~ 45 kg) and end (~ 110 kg) of the test, with an intermediate weight of ~ 80 kg. In addition, when average weight in the pen was near 110 kg (actual weight 112 ± 11.05 kg, actual age 155 ± 7.27 d), boars were individually weighed and depths of backfat (FAT) and *longissimus* muscle (LMD) were measured approximately 7 cm off the midline over the last three ribs using ultrasound (Aloka 500, Imagomedical Inc., QC) and Biotronics Toolbox Software (Biotronics Inc., Ames, IA).

Individual meal events were edited to remove outliers and obvious errors using adapted procedures recommended by Casey et al. [30], as described in [29]. All boars had to have a minimum of 63 valid feed intake days to pass the edits, along with a minimum of two valid feed intake days per week while on test. Following these edits, daily feed intake was calculated as the sum of individual feed intake events per day. Average daily feed intake (ADFI) was calculated as the predicted feed intake at the midpoint age on test for each boar based on intra-pig linear regression of daily feed intake on age. Average daily gain (ADG) was calculated using linear regression of weight on age using the weights recorded at the start and end of test, along with one or two intermediate weights, with a minimum of two weeks between any two weight records. All phenotypic records (ADFI, ADG, FAT, and LMD) were further edited by removing observations that were more than three standard deviations from their respective means. After editing, all traits followed a normal distribution and were used for further analysis.

### Variant genotyping and imputation

Genomic DNA was isolated from tail tissue samples following the DNA Extraction instruction manual (Thermo Fisher Scientific Ltd., Ottawa, ON, Canada). Samples from all animals (1363) with phenotypic records were genotyped using the Geneseek-Neogen GPPHD 80K SNP chip. A deep pedigree for these animals was traced back ~ 8 generations. The common ancestors and their genetic contribution to the studied population (1363) were calculated using the PEDIG program [31]. On the basis of “the proportion of genetic diversity” strategy, as suggested by Druet et al. [14], the top 29 ancestors (22 boars and 7 sows) based on their genetic contributions to the 1363 evaluated animals that had available tissue samples, were selected for next-generation sequencing (with an average 12-fold coverage). These ancestors cumulatively contributed about 70% of the genetics of the studied population. To improve imputation accuracy, 171 animals were genotyped with the Affymetrix Axiom^®^ 650K SNP Array, including: (1) the 94 sires, maternal grand-sires/great-grandsires of the 1363 animals, (2) the 29 sequenced animals, (3) 19 sons of the sequenced animals, and (4) the next 29 ancestors (19 boars and 10 dams), which cumulatively contributed about 20% of the genetics of the studied population. In order to test the accuracy of imputed genotypes across three different genotyping platforms, the 29 sequenced animals and 67 of the animals with 650K genotypes were also genotyped with the 80K SNP chip. All genotyping and sequencing analyses were conducted by Delta Genomics (Edmonton, AB, Canada). Library construction for next-generation sequencing was performed with 1 μg of genomic DNA according to library preparation protocols (Bio-O Scientific NEXTflex™ DNA Sequencing Kit). The Illumina 100 paired-end sequencing kit was used for sequencing on an Illumina HiSeq 2000 PE100. Variant calling was performed according to GATK Best Practices work flow [32, 33]. More specifically, Illumina reads were aligned to the reference genome (*Sscrofa 10.2*) using BWA [34]. Then, duplicates were marked and GATK INDEL realignment [35] and base quality score recalibration were applied. After that, we performed variant calling with HaplotypeCaller and joint genotyping on all samples. Finally, SNPs and Indels were filtered using parameters recommended by GATK Best Practices [32, 33].

A total of 16,560,854 autosomal variants were detected in the 29 sequenced animals, including 2,576,543 Indels and 13,984,543 SNPs. Before imputation, alleles for all SNPs on the 80K and 650K panels were converted to the standard reference (*Sscrofa 10.2*), with the reference-based allele denoted 0 and the alternate allele denoted 1. SNPs or variants for each genotyping platform were filtered for analysis according to the following criteria: SNP or variant call rate higher than 95%, SNP or variant with map information on autosomes (*Sscrofa 10.2*), Chi square of Hardy–Weinberg equilibrium test less than 600, and minor allele frequency (MAF) in the genotyped animals higher than 5%. Stepwise imputation from 80K to 650K and then to the whole-genome sequence was performed by Fimpute v2.2 [36] with inclusion of pedigree information. Leave-one-out cross-validation using the 96 animals that had both 80K and 650K genotypes and the 29 animals that had both 650K and whole-genome sequence genotypes was used to evaluate the imputation accuracy in each step. Only SNPs or variants with an imputation accuracy higher than 95% were used for further analysis. Genotype imputation accuracy was defined as the percentage of correctly imputed genotypes among the animals. Finally, 38,440 SNPs remained from the 80K panel, 429,130 SNPs remained from the 650K panel, and 4,844,535 variants were contained in the imputed whole-genome sequence.

### Genomic evaluation

#### Phenotype correction and estimation of breeding values

Significance of all possible systematic effects on phenotype, including the fixed effects of contemporary group (78 levels) consisting of ultrasonic test date and grow-finish pen, ultrasonic test machine (two levels, for FAT and LMD only), and the covariate of animal age at the end of the test (140 to 170 days), were tested using the following univariate animal model in ASREML [37]:

1 $${\mathbf{y}} = {\mathbf{Xb}} + {\mathbf{Za}} + {\mathbf{e}},$$

where **y** is the vector of observations for the trait, **b** is a vector of fixed effects (contemporary group and machine) and covariate (age), **a** is a vector of random additive genetic effects [${\mathbf{a}}\sim{\text{N}}\left( {{\mathbf{0}}, {\mathbf{A}} \times \sigma_{a}^{2} } \right)$], where ${\mathbf{A}}$ is the additive genetic relationship matrix constructed using pedigree and $\sigma_{a}^{2}$ is the additive genetic variance, ${\mathbf{e}}$ is a vector of random residuals [${\mathbf{e}}\sim{\text{N}}\left( {{\mathbf{0}},{\mathbf{I}} \times \sigma_{e}^{2} } \right)]$, where ${\mathbf{I}}$ is the identity matrix and $\sigma_{e}^{2}$ is the residual variance, and ${\mathbf{X}}$ and ${\mathbf{Z}}$ are incidence matrices associating ${\mathbf{b}}$ and ${\mathbf{a}}$ with ${\mathbf{y}}$. Only significant (*P* < 0.01) fixed effects were included in the final model to estimate the variance components and residuals of the traits. The effects of contemporary group and animal age were significant for all traits, and ultrasonic test machine was significant for FAT and LMD. The interaction between contemporary group and ultrasonic test machine was not significant. Corrected phenotypes were calculated as the sum of the estimated breeding value and the estimated residuals from the above univariate pedigree-based animal model.

Then, the 1363 Duroc boars were split into training (n = 1167) and prediction datasets (n = 196) based on birthdate, before and after June 10, 2014, respectively. The 196 youngest animals for prediction were from 19 sires and 88 dams, and almost all had half-sibs in the training dataset. The genetic relationship between individuals in the training and prediction datasets averaged 0.11 based on pedigree data. First, a full animal model (all available phenotypes) was used to obtain estimated breeding values (EBV), i.e. EBV1, and corrected phenotypes (${\text{y}}_{{{\text{c}}1}}$) for the validation animals. These ${\text{y}}_{{{\text{c}}1}}$ were used to measure the accuracy and bias of all prediction models. Second, a reduced animal model (masking the phenotypes of validation animals) was used to calculate the EBV (EBV2) of the validation animals and corrected phenotypes (${\text{y}}_{{{\text{c}}2}}$) of training animals. These ${\text{y}}_{{{\text{c}}2}}$ of training animals were used as pseudo-phenotypes in the BayesRC method (see below) to estimate the effect of SNPs. The resulting EBV2 of validation animals were then used to evaluate the pedigree-based prediction ability (BLUP method below).

#### Pre-selection, biological priors and classification of whole-genome sequence variants

The top variants (SNPs and Indels) were selected from the imputed whole-genome sequence data based on their effects on phenotype, as estimated in the training dataset (n = 1167) using method BayesB in GenSel [27, 28]. The following model was used:

2 $${\mathbf{y}} = {\mathbf{Xb}} + \mathop \sum \limits_{{\mathbf{j}}}^{{\mathbf{k}}} {\mathbf{z}}_{{\mathbf{j}}} {\upalpha}_{\text{j}} {\updelta}_{\text{j}} + {\varvec{\upvarepsilon}},$$

where ${\mathbf{y}}$ is the vector of observations for the traits, ${\mathbf{b}}$ is a vector of the significant fixed effects and covariate, as described in Eq. (1), ${\mathbf{z}}_{{\mathbf{j}}}$ is the vector of genotype covariates (− 10/0/10) across animals for SNP $j$ ($j$ = 1 to $k$), α_j_ is the allele substitution effect for SNP $j$, and δ_j_ is an indicator for whether SNP $j$ was included (δ_j_ = 1) or excluded (δ_j_ = 0) in the model for a given Markov chain Monte Carlo (MCMC) iteration. A total of 50,000 iterations were run for each analysis, with the first 5000 iterations used as burn-in. The prior probability of a SNP to have no effect was set equal to π = 0.9995 based on the posterior value obtained from BayesCπ. Due to the computational demands associated with testing the very large number of sequence variants (~ 4.8 × 10^6^) simultaneously, an alternative split-and-merge method was used, similar to Calus et al. [17]. Briefly, on each chromosome, the sequence-based variants were extracted and merged with the SNPs from the 80K SNP panel on the other chromosomes to generate sub-datasets (n = 18). The association analysis was then conducted separately on each sub-dataset using BayesB. Subsequently, results from all sequence variants across all chromosomes were combined and ordered according to the absolute value of the estimated marker effect from highest to lowest for each trait. The top 0.05% (equal to 1 − π) of variants were considered to have an important effect on the trait and selected as markers that were given a different prior for sequence variant classification (see below). Finally, 7855 markers were selected for the four traits (2025 for each trait, with 245 shared between at least two traits).

The imputed whole-genome variants were annotated based on the *Sscrofa 10.2* assembly of the swine genome using NGS-SNP [38]. All variants were then defined as belonging to one of three broad categories, as suggested by MacLeod et al. [19]. The first category, which will be referred to as “NSC”, comprised variants that were statistically associated with the traits (preselected from genome-wide association analyses (GWAS), as described above) and variants predicted to cause a non-synonymous coding change, including missense variants, splice site variants, in-frame Indels, frame shift variants, and stop gained/lost mutations. The second category, referred to as “REG”, included variants in regions that were predicted to have potential regulatory roles, mainly those within 5000 bp upstream and downstream of genes, variants in the 3′ or 5′ untranslated genic regions, and non-coding exon variants. All other variants were allocated to the third category, referred to as “CHIP”. These were mainly intergenic but included some intronic and synonymous coding variants. Then, the imputed whole-genome sequence variants were further filtered based on LD using PLINK [39] by excluding a random variant of a pair of variants that were in complete LD (*r*^*2*^ > 0.99) in a 5000-kb sliding-window with 50 variants. LD pruning was carried out first independently within each category (NSC, REG and CHIP) and then any REG or CHIP variant that was in complete LD with an NSC variant was removed. Finally, all CHIP variants that were in complete LD with a REG variant were removed. The remaining 2,154,844 variants, henceforth referred to as “SEQ”, were used for genomic prediction. They included 13,642 NSC, 157,809 REG and 1,983,393 CHIP variants.

#### Genomic prediction

Genomic predictions for the validation animals were estimated based on their genotypes (38,440 from 80K, 429,130 from imputed 650K and 2,154,844 from SEQ) and the marker effects estimated in the training dataset using three methods: GBLUP [26], BayesB [27, 28] and BayesRC [19] (the latter was only used for “SEQ”). Accuracy of prediction was evaluated by correlating the genomic breeding value of the validation animals with their corrected phenotype and dividing by the square root of the heritability of the trait. Bias of genomic predictions was estimated as the linear regression of predictions on corrected phenotypes for the validation animals, with a regression coefficient equal to 1 indicating no bias. Corrected phenotypes used for validation were obtained from analysis of the full dataset using the model of Eq. (1), as the sum of the pedigree-based EBV1 and residuals. The accuracy of genomic predictions was compared to the accuracy of pedigree-based predictions of the validation animals, which were obtained by fitting the model of Eq. (1) to the dataset with phenotypes for validation animals masked.

#### GBLUP

The genomic relationship matrix (${\mathbf{G}}$**)** based on each of the three sets of genotypes was calculated using PLINK. The GBLUP approach was applied to the model of Eq. (1), but using the genomic relationship matrix ${\mathbf{G}}$, instead of the pedigree-based relationship matrix, and with the phenotypes of validation animals masked.

#### BayesB

In the Bayesian approach, first the fraction of loci with no effect, $\pi$, was estimated using method BayesC $\pi$ in GenSel, using the full dataset. The posterior mean of $\pi$ was similar for all traits, at approximately 0.99, 0.999, 0.9995 for the 80K, 650K and SEQ genotypes, respectively. Then, the BayesB method using the model of Eq. (2) was applied to genotypes and phenotypes of the training dataset with the corresponding estimates of $\pi$ to simultaneously estimate effects of SNPs across the entire genome for the 80K, 650K and SEQ genotypes. The total number of iterations was 80,000, with 10,000 discarded as burn-in. Then the genomic prediction for the animals in the validation dataset were computed as in Eq. (3):

3 $${\text{GEBV}}_{i} = \mathop \sum \limits_{j = 1}^{k} z_{ij} \hat{\alpha}_{j},$$

where ${\text{GEBV}}_{i}$ is the genomic EBV for validation animal $i$, $j$ = 1 to $k$ is the number of SNPs in the respective genotype datasets, $z_{ij}$ is the SNP genotype code (− 10/0/10) for validation animal $i$ for SNP $j$, and $\hat{\alpha }_{j}$ is the effect estimate for SNP $j$ obtained from BayesB according to Eq. (2).

#### BayesRC

BayesRC was applied to the SEQ variants only, following MacLeod et al. [19]. Briefly, BayesRC uses an MCMC approach to estimate variant effects that are modelled as a mixture of four normal distributions, including a null distribution, $N\left( {0, 0.0 \times \sigma_{g}^{2} } \right)$, and three others: $N\left( {0, 0.0001 \times \sigma_{g}^{2} } \right)$, $N\left( {0, 0.001 \times \sigma_{g}^{2} } \right)$, $N\left( {0, 0.01 \times \sigma_{g}^{2} } \right)$, where $\sigma_{g}^{2}$ is the additive genetic variance for the trait based on whole-sequence genotypes. The first distribution accommodates the likelihood that many variants have no effect on the trait, thus reducing the complexity of the model. The model fitted to the datasets was:

4 $$\bf {\text{y}}_{{{\text{c}}2}} = 1{\varvec{\upmu}} + {\mathbf{Za}} + {\mathbf{Wv}} + {\mathbf{e}},$$

where $\bf {\text{y}}_{{{\text{c}}2}}$ is the corrected phenotype for the trait, ${\mathbf{Z}}$ is the design matrix allocating phenotypes to polygenic breeding values, ${\mathbf{a}}$ is the vector of polygenic breeding values [$N\left({{\mathbf{0}}, {\mathbf{A}} \times \sigma_{a}^{2}} \right)$], with ${\mathbf{A}}$ as the genetic relationships calculated from pedigree and $\sigma_{a}^{2}$ as the additive genetic variance not explained by the variants, ${\mathbf{W}}$ is the design matrix of variant genotypes (0/1/2), centred and standardized to have unit variance, ${\mathbf{v}}$ is the vector of estimated variant effects based on a mixture of the four distributions as listed above, and ${\mathbf{e}}$ is the vector of random residuals.

Prior independent biological information was used to allocate each variant to a “class” $c$ ($c$ = 3), as described above, where the purpose is to provide one or more classes that are expected to be enriched for QTL or for variants linked to the QTL. As described by Macleod et al. [19], within each class *c*, a uniform *Dirichlet* prior was used for the proportion of effects in each of the four normal distributions of SNP effects.

For all traits, we implemented five replicate chains of 80,000 iterations of the Gibbs sampler, with 10,000 iterations discarded as burn-in. Very good agreement was found in the final results across the five replicate chains (correlation of posterior estimates of marker effects equal to 0.999). Final estimates were derived from the means of the five replicate chains. Using the resulting posterior means of marker effects, the genomic breeding value for the validation animals were calculated using Eq. (3).

## Results

### Genotype imputation accuracy

The average genotype imputation accuracy for individual SNPs was 92.1% from 80K to 650K and 85.6% from 650K to whole-genome sequence, with the complete range from 0 to 100% across SNPs. Most SNPs had an imputation accuracy higher than 90%, 77% of SNPs for imputation from 80K to 650K and 57% for imputation from 650K to sequence. About 12.6 and 25.9% of SNPs had an imputation accuracy lower than 80% for imputation of 80K–650K and of 650K to sequence imputation, respectively (Table 1). Only variants with an imputation accuracy higher than 95% were kept for final genomic prediction.

**Table 1:** Percentage of SNPs in different ranges of imputation accuracy from 80K to 650K and 650K to sequence

Range of imputation accuracy (%)	80K to 650K	650K to sequence
< 80	12.6	25.9
80–85	4.2	5.3
85–90	6.2	11.4
90–95	11.8	10.5
> 95	65.2	46.9

### Genomic prediction accuracy

#### Genomic prediction versus pedigree-based prediction

The accuracy and bias of (G)EBV for the studied traits are in Table 2. Generally, the average accuracy of GEBV was moderate to high for ADFI (0.40) and FAT (0.65), and relatively low for LMD (0.30) and ADG (0.15). Compared with the pedigree-based evaluation, the use of genomics was beneficial for ADFI and FAT, with smaller bias and an accuracy that was improved by on average 42.9 and 32.7%, respectively. However, for ADG and LMD, pedigree-based prediction gave better accuracy and smaller bias, and no improvement was observed from using genomic data.

**Table 2:** Accuracy and bias of (G)EBV evaluated using pedigree, 80K, 650K and SEQ data using different prediction methods

Resource	Method	ADFI		FAT		ADG		LMD	
		Accuracy	Bias	Accuracy	Bias	Accuracy	Bias	Accuracy	Bias
Pedigree	BLUP	0.28	0.83	0.49	0.91	0.28	0.53	0.42	1.17
80K	GBLUP	0.38	0.96	0.66	0.98	0.17	0.31	0.29	0.63
	BayesB	0.44	1.14	0.68	1.12	0.12	0.23	0.25	0.59
650K	GBLUP	0.38	0.99	0.64	0.95	0.20	0.38	0.29	0.6
	BayesB	0.45	1.17	0.68	1.17	0.09	0.15	0.26	0.59
SEQ	GBLUP	0.37	0.95	0.59	0.96	0.12	0.28	0.32	0.69
	BayesB	0.41	1.07	0.65	1.27	0.12	0.21	0.32	0.77
	BayesRC	0.40	0.64	0.62	0.81	0.25	0.32	0.35	0.64
Average accuracy of using genomic data		0.40	0.97	0.65	1.02	0.15	0.30	0.30	0.71

*ADFI* average daily feed intake, *FAT* ultrasound backfat depth, *ADG* average daily gain, *LMD* ultrasound loin muscle depth

#### Bayesian methods versus GBLUP

**Fig. 1:**
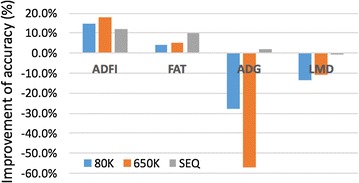
Improvement (%) of GEBV accuracy using BayesB compared with using GBLUP. The improvement was defined as 100 × (*Accuracy_BayesB* − *Accuracy_GBLUP*)/*Accuracy_GBLUP*, indicating how much improvement of accuracy using BayesB compared with using GBLUP

Improvement in the accuracy of genomic predictions based on BayesB compared with GBLUP is shown in Fig. 1. Generally, BayesB performed better than GBLUP for ADFI and FAT for all three sets of genotypes (positive in Fig. 1). For ADG and LMD, GBLUP gave higher accuracy using 80K and 650K SNPs (negative in Fig. 1), but little difference in accuracy was observed between the two methods when using SEQ data. When applied to the SEQ data, BayesRC resulted in higher accuracy than BayesB and GBLUP for both ADG and LMD. For ADFI and FAT, the accuracy from BayesRC was between those from GBLUP and BayesB (Table 2).

#### Accuracy from different marker densities

**Fig. 2:**
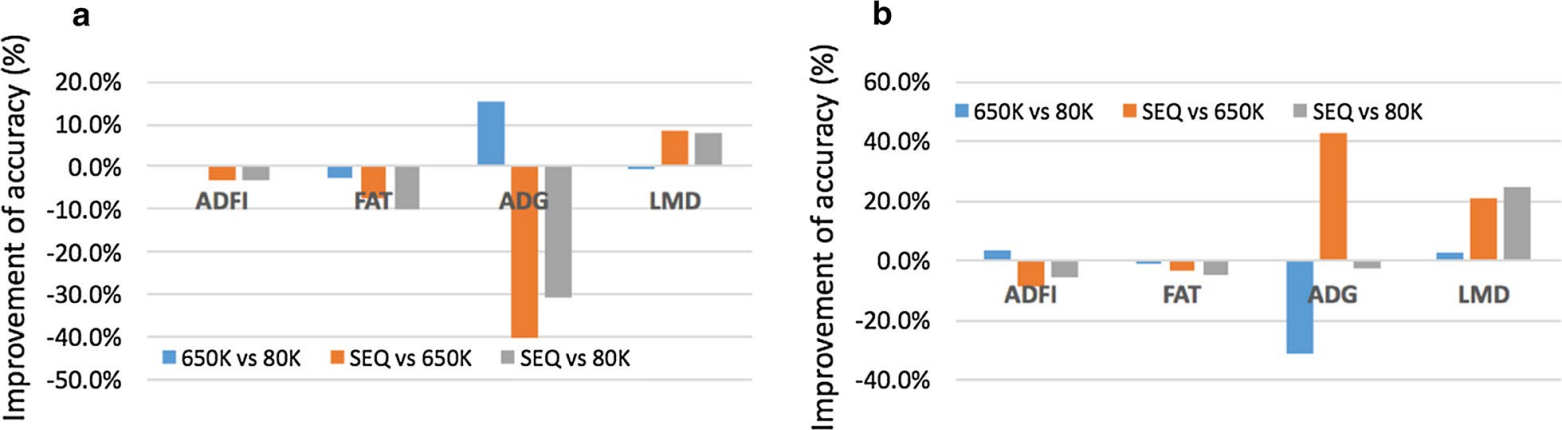
Improvement (%) of GEBV accuracy with increasing marker density. Improvement was defined as 100 × *(Accuracy_higher*-*density* − *Accuracy_lower*-*density)/Accuracy_lower*-*density*, indicating how much improvement of accuracy from low to high marker density. **a** Using GBLUP method, **b** using BayesB method

The change in the accuracy of genomic predictions with increasing SNP density is in Fig. 2. Increasing the SNP density slightly decreased the prediction accuracy for FAT, for which use of 80K SNPs gave the best accuracy regardless of the statistical method used. For ADFI, use of SEQ data decreased the accuracy compared with the SNP panels, and little difference in accuracy was observed between 80K and 650K. For LMD, increasing the number of SNPs resulted in similar or greater accuracy for both GBLUP and BayesB. For ADG, almost no improvement in accuracy was observed with increasing marker density. In conclusion, SEQ data with the BayesRC method gave the best accuracy for ADG (0.25) and LMD (0.35), while use of 80K SNPs with the BayesB method gave the best accuracy for ADFI (0.44) and FAT (0.68).

## Discussion

### Genotype imputation

In the current study, we obtained high imputation accuracies, which reached 0.92 for imputation from 80K to 650K SNPs. In pigs, there are several reports on the accuracy of genotype imputation from lower densities to 60K SNPs, with correlations between observed and imputed genotypes ranging from 0.952 to 0.995 for imputation from 6K to 60K [40], from 0.879 to 0.991 for imputation from 3K or 6K to 60K [41, 42], and from 0.88 to 0.95 for imputation from 9K to 60K in different scenarios [9]. However, imputation using high-density and whole-genome sequence using pig data has not been reported to date. In beef cattle, the percentage of correctly imputed genotypes was on average 95% for imputation from 3K to 50K [43] and ranged from 84 to 99% for imputation from 50K to 777K [24, 44]. The squared correlation (*R*^*2*^) between imputed and observed genotypes ranged from 0.80 to 0.96 for imputation from different low densities to 50K [45] and from 0.90 to 0.96 for imputation from 50K to 777K [46]. For sequence imputation, research in Holstein–Friesian bulls has shown that stepwise imputation (50K–777K to sequence) yields higher accuracy (correlation between observed and imputed genotypes) (0.77 to 0.83) than using a one-step method, which had accuracies ranging from 0.37 to 0.46 for imputation from 50K to sequence and from 0.77 to 0.83 for imputation from 777K to sequence [47]. Imputation accuracy measured as the percentage of variants correctly imputed was on average 85.6% to whole-genome sequence data in the current study, which was lower than that obtained in Holstein cattle (97%) with a large multi-breed reference population (n = 444) [48].

Many factors can influence the accuracy of genotype imputation. MAF is one important factor, especially when imputing to sequence data, since the number of SNPs with a very low MAF is usually limited in SNP panels but large in sequence data (see Additional file 1: Fig. S1). The effect of MAF on accuracy was even greater when the accuracy was measured as the percentage of correctly imputed variants (the measurement applied here) than measured by other statistics. In cattle, SNPs with a very low MAF had very poor imputation accuracy, which heavily influenced the overall imputation accuracy [47], especially when the reference population (founders) was small [16, 49], as is the case in this study, where only 29 common ancestors were sequenced as the reference population. Relationships between individuals is another factor that affects imputation accuracy. In general, imputation accuracy increases with lower relatedness within the reference population and larger relationships between reference and imputed individuals. As reported previously, a multi-breed reference population generated higher imputation accuracy for a given breed than using the same breed as a reference [23, 50]. In the present study, in order to maximize relationships between reference and imputed animals, 29 common ancestors that contributed about 70% of the alleles present in the imputed animals were selected for sequencing. However, as we were restricted to one breed and in availability of tissue samples, the selected animals (29) in the reference population were related to each other, with genetic relationships ranging from 0.03 to 0.49 and averaging 0.06.

### Genomic prediction

The average accuracies of genomic predictions for ADFI, FAT, ADG and LMD obtained in this study were 0.40, 0.65, 0.15 and 0.30, respectively. Limited literature is available on genomic prediction for feed efficiency and component traits in pigs (Table 3). Studies for Duroc pigs using 60K SNPs showed accuracies of genomic predictions for ADFI, FAT, ADG and LMD of about 0.15, 0.37–0.56, 0.24–0.58 and 0.30, respectively [10–12]. A study using imputed 60K SNPs in Yorkshire pigs [9] reported accuracies of 0.69–0.86 for FAT and of 0.66–0.88 for growth rate. Accuracies of genomic predictions obtained in this study were much higher for ADFI than accuracies obtained in these previous reports but much lower for ADG, while accuracies obtained for FAT and LMD were in the range of previous reports (Table 3). These differences can be explained by the many factors that influence the accuracy of genomic prediction, which will be discussed later.

**Table 3:** Literature estimates of the accuracy of genomic predictions of feed efficiency component traits in pigs

Trait	Accuracy^a^	Breed and reference
Days to 250 lbs	0.66–0.84	Yorkshire [9]
ADG	0.50–0.58^b^	Danish Duroc [12]
	0.40–0.43^b^	Danish Duroc [10]
	0.24	Duroc [11]
Feed conversion ratio	0.39–0.45^b^	Danish Duroc [12]
	0.11	Duroc [11]
FAT	0.69–0.86	Yorkshire [9]
	0.55–0.56^b^	Danish Duroc [10]
	0.37	Duroc [11]
ADFI	0.15	Duroc [11]
Residual feed intake	0.09	
LMD	0.30	

^a^Correlation of genomic predictions and corrected phenotype divided by square root of heritability, which was also used in our study; ^b^converted from the reliability reported in the literatures

*ADFI* average daily feed intake, *FAT* ultrasound backfat depth, *ADG* average daily gain, *LMD* ultrasound loin muscle depth

The advantage of using genomic information for breeding value prediction over using pedigree information (BLUP method) was not uniform across traits. Compared to pedigree-BLUP, using genomic data increased prediction accuracy and decreased prediction bias for ADFI and FAT but not for ADG and LMD. Similar results were reported in cattle [51] and sheep [23], where the use of genomic data for genomic prediction was not beneficial for all traits. Use of genomic information is generally expected to increase prediction accuracy, such as the reports in chicken [18, 52] and pigs [53], since genomic data can consider the Mendelian sampling terms better compared with pedigree information, and can produce more accurate genetic relationships among animals. However, this is not always true, as discussed above. The other two main factors that affect genomic prediction accuracy are the ability of markers to capture the total genetic variance of the traits (so-called “genomic heritability”) and the accuracy of the estimates of marker effects [54]. In most cases, heritability estimates obtained from dense markers were lower than estimates obtained from pedigree-based animal models (see Additional file 2: Table S1), which indicates that “missing heritability” exists, and this has been reported to be an issue in human genetics [55, 56]. Missing heritability mainly results from incomplete LD between causal variants and genotyped SNPs, which can be exacerbated by causal variants having lower MAF than the genotyped SNPs [55, 56]. Missing heritability can also be related to the genetic architecture of the traits, epistatic effects, genotype-by-environment interactions, and others [57]. For example, if the SNPs used are causal variants or are closely-linked to causal variants for the traits, they can capture a large proportion of the genetic variance and give high genomic prediction accuracies, such as for ADFI and FAT in this study, for which QTL with relatively large effects have been detected (data not shown). If the SNPs used do not capture all the genetic variation for the trait, prediction accuracy is limited, such as the low prediction accuracy found for ADG and LMD, for which the SNP panels only captured 53 to 83% of the genetic variance based on pedigree- and genotype-based estimates of heritability (see Additional file 2: Table S1). A similar trend was also reported in sheep [23], where no significant regions or markers were detected for the two traits for which prediction accuracy was not increased by using genomic data compared with using pedigree information.

#### Genetic architecture of traits and genomic prediction method

Genetic architecture and the statistical method used for genomic prediction are two interrelated factors that have a large influence on the accuracy of genomic prediction. Usually, higher accuracy can be achieved when the model assumptions more closely represent the underlying genetic architecture of the traits. We found that BayesB outperformed GBLUP in the accuracy of genomic prediction for ADFI and FAT. BayesB assumes that only a small proportion of SNPs have a large effect on the trait, which is in agreement with our GWAS results, where relatively large QTL were detected on *Sus scrofa* chromosomes (SSC) SSC1 and SSC18 for ADFI and FAT, using BayesB in the full dataset (data not shown). With this method, the effects of SNPs surrounding large QTL, such as those on SSC1 and SSC18 for ADFI and FAT, are easier to detect and more accurately estimated. This could be the main reason why BayesB gave higher accuracy for ADFI and FAT than GBLUP. The advantage of BayesB for genomic prediction for traits that are, at least in part, determined by QTL of large effect was also recognized by Meuwissen et al. [58] and demonstrated by other empirical studies [5, 22, 46, 59, 60]. For ADG and LMD, GBLUP performed better and increased the accuracy by 3 to 11% compared to BayesB. GBLUP assumes an infinitesimal model and, thereby, that all markers have the same contribution to the trait (e.g. no major QTL control the trait). Compared with FAT, few QTL were detected for ADG (data not shown), indicating that ADG may be determined by many loci with very small individual effects.

We also implemented the BayesRC method for the imputed whole-genome sequence data. Compared to BayesB and GBLUP, BayesRC gave higher accuracy for ADG and LMD, improving accuracy from 0.12 to 0.25 for ADG and from 0.32 to 0.35 for LMD. For ADFI and FAT, the accuracy from BayesRC was between those obtained with GBLUP and BayesB. The advantage of BayesRC compared with GBLUP and BayesB is that it can incorporate prior biological information by defining classes of variants that are likely enriched for causal mutations and by fitting a mixture distribution for the effects of variants in each class [15, 19, 61], which is more precise and sensitive to the genetic architecture of the traits. BayesRC resulted in the most accurate genomic predictions for ADG and LMD but also introduced greater bias for ADFI and LMD (Table 2). Both simulation and empirical studies have also shown that BayesRC can increase the power of detection of causal variants and improve the accuracy of genomic prediction compared to GBLUP [19, 46], in agreement with this study. BayesRC is also able to detect a larger proportion of variance when there is a large number of QTL with small individual effects [62], as was the case for ADG in this study. The posterior π values for class NSC that was obtained for ADG (0.413) was much smaller than the posterior π for FAT (0.641), which indicates that a larger proportion of variants were in class NSC for ADG and these variants may have small individual effects on the trait. Therefore, for traits with such a genetic architecture (e.g. ADG), the advantage of BayesRC for genomic prediction is greater. For traits with known large QTL, such as ADFI and FAT, accuracies obtained with BayesB and BayesRC were similar or higher than those obtained using GBLUP. However, the advantage of BayesRC for sequence data depends on the completeness and accuracy of the prior biological information. With a better understanding of the functional annotation of genes and variants in the future [63], the benefit of using whole-sequence data for genomic prediction is anticipated to be further improved.

#### Impact of marker density on genomic prediction

Increasing marker density has the potential to improve the accuracy of genomic prediction and the use of whole-genome sequence data is expected to give the best accuracy, as the causative mutations are expected to be included in the genotype data [16, 64]. However, this was not found to be always the case in our study. An increase in marker density did not improve prediction accuracy for some traits, such as FAT, for which 80K SNPs gave the highest accuracy, regardless of the statistical method used. This result was also reported for backfat thickness in pigs by Pérez-Enciso et al. [62]. Similar results were also obtained in cattle, for which imputed 777K SNPs resulted in no or very little increase in the accuracy of genomic prediction for some traits [17, 65–67] compared with using 50K SNPs. SNPs on commercially available low-density SNP chips (e.g. pig 60K and 80K, bovine 50K) were selected to have a high MAF and can, thus, capture a relatively large amount of the variance for traits that are determined by a relatively small number of QTL (e.g. backfat in pigs). Increasing marker density has little effect on capturing the remaining proportion of genetic variance for such traits. Furthermore, with GBLUP and BayesB, the QTL effects and opportunities for their detection become smaller with increasing density [16], thus resulting in less accurate genomic predictions. Therefore, we suggest that the 80K SNP panel is sufficient for within-breed genomic prediction for FAT and yields acceptable accuracy (0.68). In contrast, when some of the QTL mutations or the linked SNPs are not in the SNP panel, a higher density may include more SNPs that are in high LD with the QTL for the traits, resulting in an increase in the genetic variance captured and more accurate genomic predictions. This appeared to be the case for ADG in this study. As discussed above, when considering the best method for each trait, the imputed 650K SNPs increased the accuracy of genomic prediction by 3.4% for ADFI (BayesB) and 15.2% for ADG (GBLUP).

Results from using sequence data to improve the accuracy of genomic prediction have been inconsistent. Simulation studies suggested that including whole-sequence data could improve the accuracy of genomic prediction by as much as 40%, depending on the trait, statistical method, and MAF of the causal mutations affecting the trait [14, 16, 25, 68]. However, empirical studies in cattle and chickens have reported either no or a very small increase in accuracy when using imputed whole-genome sequence data compared to using the available low- or high-density SNP chips [17, 18, 21, 64, 69]. In pigs, simulation based on whole-genome sequence showed an increase in accuracy of ~ 3.8% over 60K and ~ 2.8% over 650K SNPs [62]. We found that using SEQ data and BayesRC gave the highest prediction accuracies for LMD and ADG. For LMD, using SEQ data increased the accuracy from 8 (GBLUP) to ~ 20% (BayesB). Using SEQ data, however, resulted in a decrease in accuracy for FAT and ADFI compared to using 80K and 650K SNP chips. Druet et al. [14] explained that the advantage of using imputed sequence data for genomic prediction is affected by the accuracy of imputation and, more importantly, by the allele frequency distribution of the QTL. When the MAF of QTL is very low, genomic predictions from imputed sequence data can result in up to 30% improvement in accuracy. However, for rare variants, imputation accuracy is usually poorer than for variants with a high MAF [14, 47, 50]. Thus, a large reference population must be sequenced to improve the results. In this study, the small number of sequenced animals may have influenced the accuracy of imputed sequence variants and, thus, may have limited the potential of whole-genome sequence data to improve prediction accuracy.

#### Pre-selection and prior biology of sequence variants

A significant challenge for genomic prediction using whole-genome sequence data is the computational requirement due to the large number of markers. Preselecting the most important markers and/or filtering out the uninformative ones can address this problem. The split-and-merge approach, which splits one large computational task into many smaller ones, was first proposed by Calus et al. [17] to pre-select the most important markers from whole-genome sequence data. Some studies [48, 70–72] showed that using preselected markers from sequence data through GWAS and adding them to the 50K SNP panel can increase the accuracy of genomic prediction by up to 5 percentage points. However, Veerkamp et al. [73] and Calus et al. [17] found no improvement in accuracy using a similar approach. In our study, first a modified split-and-merge approach was used by integrating the 80K SNPs into the split association analyses for each chromosome, in order to better account for polygenic effects and to improve the accuracy of estimates of marker effects. Second, all SEQ markers, not only the pre-selected ones, were considered simultaneously in the genomic prediction model (BayesRC), which may avoid the loss of the marginal genetic variance contributed by the non-selected sequence variants and the possible bias derived from strict pre-selection, as discussed by Calus et al. [17] and Veerkamp et al. [73]. Pruning SNPs that are in complete and high LD with other SNPs is also an efficient way to reduce the number of uninformative markers, which was shown to be important for the application of Bayesian models that explicitly estimate a SNP variance component using sequence data, since performance of these models may be poorer without pruning [17].

#### Other factors affecting genomic prediction accuracy

According to Goddard [54], prediction accuracy depends on both the proportion of genetic variance that can be captured by markers (so-called “genomic heritability”) [74] and the accuracy of estimates of marker effects. However, there are important trade-offs between these two factors. Usually the estimate of genomic heritability increases when more markers are used, especially when the added markers are in high LD with the QTL [55, 56]. A similar trend was found in our study, where the use of SEQ variants increased the genomic heritability for all traits compared with using the commonly available SNP panels (see Additional file 2: Table S1). However, the accuracy of estimates of marker effects was impaired as the number of effects to be estimated increased, which is mainly due to the relatively small size of the training population (n ≪ p, where n is the number of animals in training and p is the number of markers). Additional issues can also arise as a result of the small sample size, including (1) causal mutations (usually with a small MAF) may be missed, are more easily filtered out during quality control, or are more poorly imputed to the whole population [47, 50], which decreases the value of such causal variants in the prediction process, thus negatively influencing prediction accuracy; and (2) a small amount of phenotypic data is not sufficient to detect causative mutations and to distinguish their effects from random noise. Therefore, as Meuwissen et al. [75] highlighted, a large training dataset is needed to take full advantage of high-density markers (especially for whole-genome sequence data) for accurate genomic prediction.

Relatedness between individuals is also very important for both genotype imputation and genomic prediction. Ideally, having less related animals in a large reference population is helpful to break down high levels of LD, thus making it easier to identify the causal mutations and to capture all the genetic variance. For example, use of multiple breeds in the training population to reduce their average relatedness gave more accurate genomic predictions than using the same single breed for training, especially in simulated datasets [15, 25, 64, 76, 77]. In contrast, greater relationships between training and prediction animals can improve the prediction accuracy. Macleod et al. [19] demonstrated that the accuracy of genomic prediction increased for all traits with increasing relatedness between training and prediction sets. Other studies also reported that a closer relationship between training and prediction increases the accuracy of genomic prediction [11, 52, 78, 79].

A small effective population size, which contributes to high LD [15], is another factor that influences the prediction accuracy. The effective size of pig breeding populations has been estimated to be relatively small (55 to 113 [80, 81]), so a small number of SNPs (80K) may capture most genetic variance, especially for ADFI and FAT, which may be determined by a small number of QTL with relatively large effects (at least in this population). Therefore, the potential increase in the accuracy of genomic prediction from using whole-genome sequence data is expected to be limited. A similar situation was also observed in dairy cattle [19, 24] and sheep [23] populations with small effective sizes.

## Conclusions

In conclusion, although the reference population used was small, the genotype imputation accuracies were as high as 92.1% from 80K to 650K, and 85.6% from 650K to whole sequence. Increasing marker density, however, had no or little advantage for genomic prediction for FAT and ADFI, such that the available 80K SNP panel is sufficient for these traits. BayesB resulted in higher prediction accuracy than the other methods tested for these two traits. For LMD and ADG, GBLUP gave higher genomic prediction accuracies than BayesB, and BayesRC in SEQ data gave the best prediction accuracies. However, pedigree-based BLUP outperformed all genomic methods and produced the highest prediction accuracies for ADG and LMD, likely because the SNPs captured less genetic variance for these traits than pedigree data. In the future, with decreasing costs for whole-genome sequencing, a better understanding of the functional annotation of the genome and variants [63], and larger reference population sizes, BayesRC is anticipated to be a superior method for genomic prediction and application in genetic improvement.

## Additional files

**Additional file 1: Fig. S1.** Histograms of MAF distribution for the variants from final 80K, 650K and SEQ data.

**Additional file 2: Table S1.** Variance component and heritability estimates using different information. The data provided presented the genetic variance, total phenotypic variance and estimated heritability for the traits using different information and methods.
