# Supplementary figures and images for: Genomic evaluation of feed efficiency component traits in Duroc pigs using 80K, 650K and whole-genome sequence variants
Source: Genet Sel Evol. 2018 Apr 6;50:14. doi: 10.1186/s12711-018-0387-9 (PMC5889553; doi:10.1186/s12711-018-0387-9)

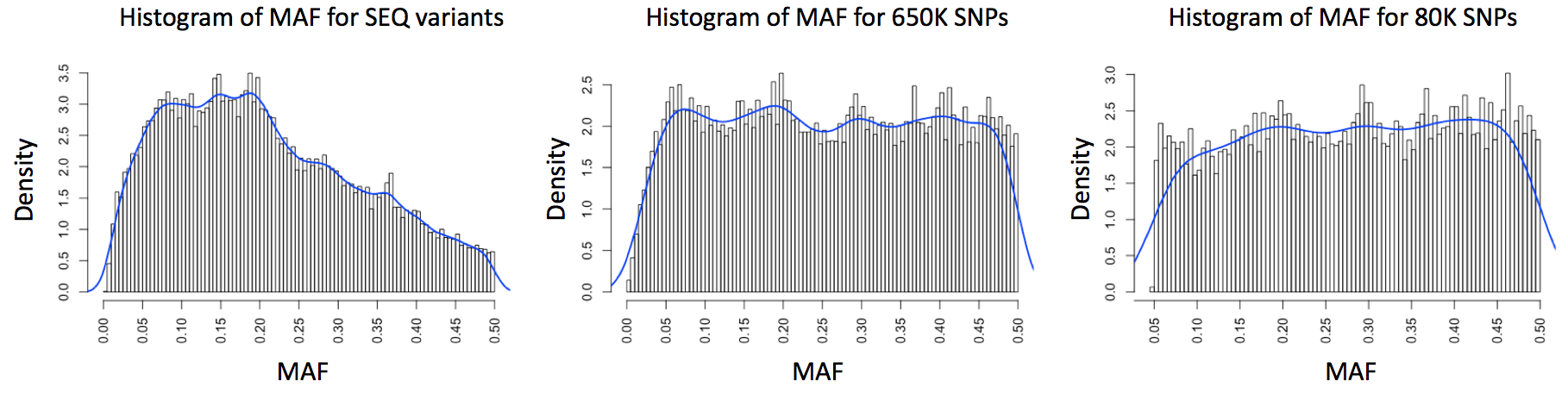

Supplement: Supplementary file 1 — Additional file 1: Fig. S1. Histograms of MAF distribution for the variants from final 80K, 650K and SEQ data. [file 12711_2018_387_MOESM1_ESM.png]
