# Supplementary material for: Genomic evaluation of feed efficiency component traits in Duroc pigs using 80K, 650K and whole-genome sequence variants
Source: Genet Sel Evol. 2018 Apr 6;50:14. doi: 10.1186/s12711-018-0387-9 (PMC5889553; doi:10.1186/s12711-018-0387-9)
Supplement: Supplementary file 2 — Additional file 2: Table S1. Variance component and heritability estimates using different information. The data provided presented the genetic variance, total phenotypic variance and estimated heritability for the traits using different information and methods. [file 12711_2018_387_MOESM2_ESM.docx]

Table S1 Variance component and heritability estimates using different information

| **Methods** | **Components** | **ADFI** | **FAT** | **ADG** | **LMD** |
| --- | --- | --- | --- | --- | --- |
| Pedigree_BLUP | GenVar | 0.03 | 3.81 | 0.003 | 9.72 |
|  | PheVar | 0.14 | 7.31 | 0.008 | 23.30 |
|  | *h^2^* | **0.21** | **0.52** | **0.34** | **0.42** |
| 80K_GBLUP/BayesB^1^ | GenVar | 0.03 | 3.42 | 0.002 | 7.98 |
|  | PheVar | 0.13 | 7.24 | 0.008 | 22.93 |
|  | *h^2^* | **0.19** | **0.47** | **0.19** | **0.35** |
| 650K_GBLUP/BayesB^1^ | GenVar | 0.02 | 3.53 | 0.001 | 7.93 |
|  | PheVar | 0.13 | 7.31 | 0.008 | 22.92 |
|  | *h^2^* | **0.18** | **0.48** | **0.18** | **0.35** |
| SEQ_GBLUP/BayesB^1^ | GenVar | 0.04 | 5.75 | 0.002 | 12.96 |
|  | PheVar | 0.15 | 9.65 | 0.009 | 28.16 |
|  | *h^2^* | **0.27** | **0.60** | **0.25** | **0.46** |
| SEQ_BayesRC | GenVar | 0.07 | 4.36 | 0.003 | 12.24 |
|  | PheVar | 0.14 | 6.70 | 0.007 | 22.16 |
|  | *h^2^* | **0.53** | **0.65** | **0.43** | **0.55** |
| Average *h^2^* of using genomic data | | **0.29 ± 0.14** | **0.55 ± 0.08** | **0.26 ± 0.10** | **0.43 ± 0.08** |

^1^Average values for those from BayesB and GBLUP, as they were similar.

ADFI: average daily feed intake; FAT: ultrasound backfat depth; ADG: average daily gain; LMD: ultrasound loin muscle depth. GenVar: genetic variance; PheVar: total phenotypic variance; *h^2^*: heritability estimate
